# Supplementary material for: Machine-Learning-Assisted Analysis of TCR Profiling Data Unveils Cross-Reactivity between SARS-CoV-2 and a Wide Spectrum of Pathogens and Other Diseases
Source: Biology (Basel). 2022 Oct 19;11(10):1531. doi: 10.3390/biology11101531 (PMC9598299; doi:10.3390/biology11101531)
Supplement: Supplementary file 1 [file biology-11-01531-s001.zip › Supplementary_Information.pdf]

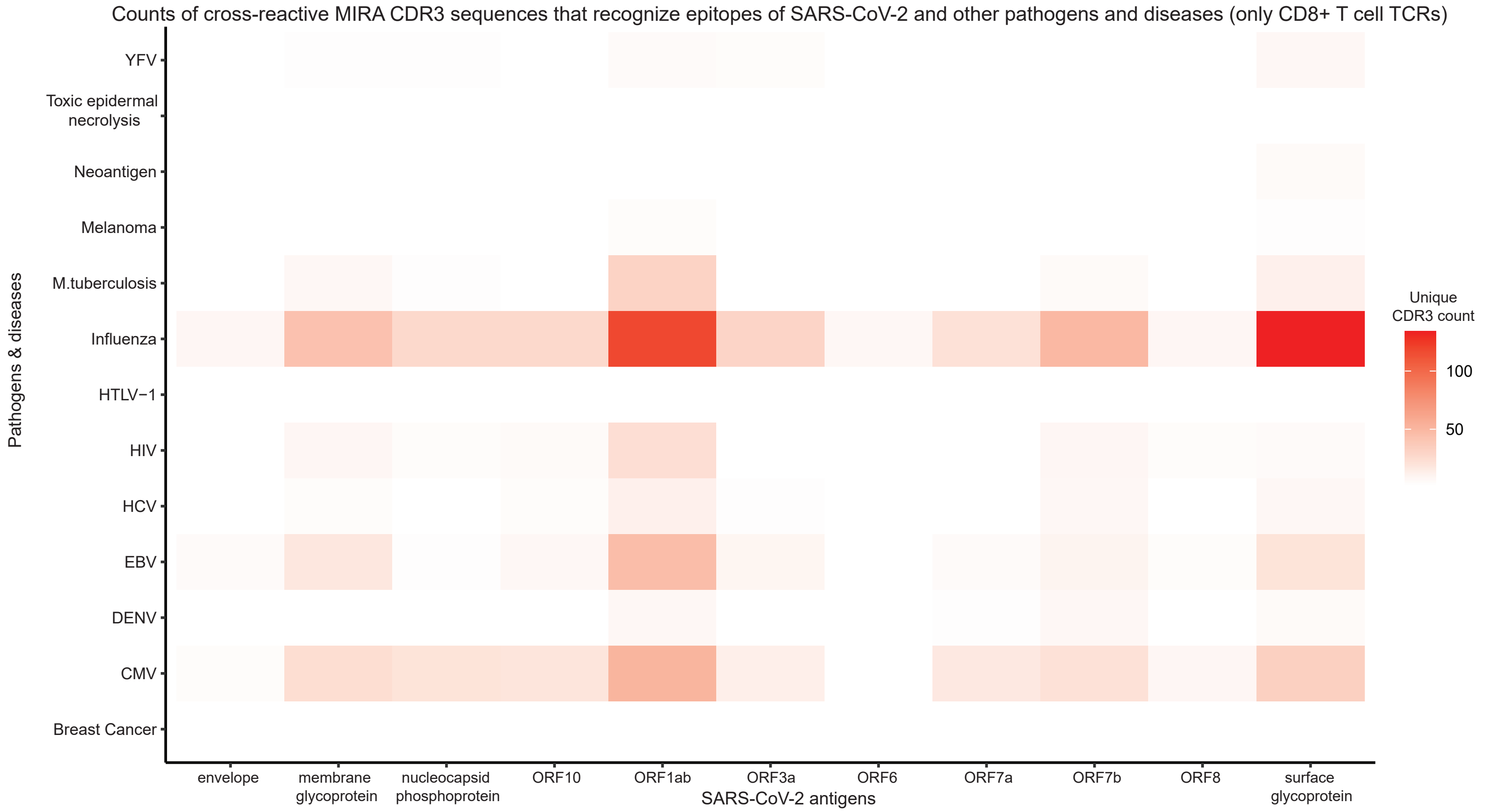
Counts of cross-reactive MIRA CDR3 sequences that recognize epitopes of SARS-CoV-2 and other pathogens and diseases (only CD8+ T cell TCRs)
YFV
Toxic epidermal necrolysis
Neoantigen
Melanoma
M.tuberculosis
Influenza
HTLV−1
HIV
HCV
EBV
DENV
CMV
Breast Cancer
Pathogens & diseases
envelope
membrane glycoprotein
nucleocapsid phosphoprotein
ORF10
ORF1ab
ORF3a
ORF6
ORF7a
ORF7b
ORF8
surface glycoprotein
SARS-CoV-2 antigens
Unique CDR3 count
100
50

Figure S1. Cross-reactivity analysis of all Multiplexed Identification of T cell Receptor Antigen (MIRA) T cell receptors (TCRs) only from CD8+ T cells. Related to Figure 5. The cross-reactivity is depicted as a heatmap of unique MIRA complementarity-determining region 3 (CDR3) counts that exhibit cross-reactivity between SARS-CoV-2 (x-axis) and other pathogens and diseases (y-axis). The heatmap values correspond to the number of unique cross-reactive CDR3 sequences rather than the frequency of occurrence across MIRA samples.
